# Forgetting Details in Visual Long-Term Memory: Decay or Interference?

**DOI:** 10.3389/fnbeh.2022.887321

**Published:** 2022-07-19

**Authors:** Laura García-Rueda, Claudia Poch, Pablo Campo

**Affiliations:** ^1^Departamento de Anatomía, Histología y Neurociencia, Universidad Autónoma de Madrid, Madrid, Spain; ^2^Facultad de Lenguas y Educación, Universidad de Nebrija, Madrid, Spain; ^3^Department of Basic Psychology, Universidad Autónoma de Madrid, Madrid, Spain

**Keywords:** episodic memory, mnemonic discrimination, pattern separation, false memory, ERPs, interference, decay

## Abstract

Two main explanations for memory loss have been proposed. On the one hand, decay theories consider that over time memory fades away. On the other hand, interference theories sustain that when similar memories are encoded, they become more prone to confusion. The interference is greater as the degree of similarity between memories increases, and as the number of similar traces increases too. To reduce interference, the pattern separation process allows the brain to separate similar memories and build detailed memory representations that are less easily confused. Nonetheless, with time, we tend to remember more general aspects of experiences, which also affects our ability to discriminate. We present the results of one experiment in which brain activity was recorded by EEG while two groups of healthy participants performed a visual memory discrimination task. This task assesses the ability to differentiate new but similar information from previously learned information and thus avoid interference. Unlike previous studies, we used a paradigm that was specifically designed to assess the impact of the number of items (2 or 6) of each category stored in memory, as well as the time elapsed after the study phase (20 min or 24 h), on recognition memory for objects. Behaviorally, our results suggest that mnemonic discrimination is not modulated by the passage of time, but by the number of stored events. ERP results show a reduced amplitude in posterior regions between 500 and 700 ms when comparing short and long delays. We also observe a more positive activity in a centro-posterior region in the 500–700 ms window at retrieval when participants store more items. Interestingly, amplitudes for old hits and similar false alarms were greater than amplitudes for correctly rejected new items between 500 and 700 ms. This finding indicates that a recollection-based process operates in both true and false recognition. We also found that the waveforms for correct rejections of similar lures and the waveforms for correct rejections of new items were comparable.

*To think is to forget differences, generalize, make abstractions. In the teeming world of Funes, there were only details, almost immediate in their presence*.

–Jorge Luis Borges (Funes the Memorious, 1944).

## Introduction

Episodic memory enables human beings to mentally time travel to past personal experiences. Despite this amazing ability, we all have experienced that, with the passage of time, our ability to remember seems to decrease. This is especially evident when we are trying to recall an episode that resembles other episodes. Gist-based false recognition occurs when we erroneously recognize an item that is similar, but not identical to a previously encountered item (Schacter et al., 2011). What happens is that we have a sense of familiarity and false memories take place. As Frederic Barlett already noted in his pioneering work, with the passage of time, there is a transition from detailed memories to more general memories (Richards and Frankland, 2017). Thus, it seems that the influence of time on forgetting of established episodic memories could be accounted by the gradual erosion and modification of memory traces, this is, by decay (Altmann and Schunn, 2012; Hardt et al., 2013; Richards and Frankland, 2017). Alternatively, another explanation sustains that forgetting is produced by interference (Wixted, 2004). Interference is viewed as a competition phenomenon, so that with the passage of time we accumulate an increasing number of similar memory traces, which during retrieval causes confusability, thus affecting the ability to recall the targeted trace (Unsworth et al., 2008; Altmann and Schunn, 2012; Yeung et al., 2013; Anderson, 2015). To reduce interference, the process of pattern separation increases memory specificity by building detailed memory representations that are less easily confused (Motley and Kirwan, 2012; Kesner, 2013; Xue, 2018). However, highly similar memories can compromise the efficiency of this process, leading to a reduced mnemonic discriminability.

Several previous studies have documented how our ability to discriminate between similar memories evolves across time. To address this question, one study (Sekeres et al., 2016) investigating time-dependent loss of “central” and “peripheral” details from episodic memories, reported that time-dependent loss of episodic memories differs in terms of detail types. This study showed that central details such as the core or gist of events showed significantly less loss than peripheral details. Similarly, Mercer and Jones (2019) observed a reduction of mnemonic discrimination between a target and a similar foil over a week delay. Brady et al. (2013) also observed a decrement in mnemonic discrimination as a function of time delay, although different object properties were affected differentially. Likewise, another study (Leal et al., 2019) used dynamic videos as stimuli, and found that after 24 h there was an increased forgetting as compared to immediate memory, which was more pronounced for detailed memory than for gist memory. By contrast, Andermane and Bowers (2015) conducted a visual long-term memory experiment following a week delay and found that detailed and gist-like visual memories decayed at similar rates.

While these studies have provided valuable information about memory discrimination between similar events over time, none of them have explored another variable thought to contribute to rate of forgetting, such as the number of similar traces stored in memory (Roediger and Agarwal, 2010; Anderson, 2015). It has been shown that the ability to discriminate between lures and old items decreases as the number of encoded items in each category increases (Konkle et al., 2010; Poch et al., 2019, 2020). Moreover, this interference phenomenon is related with semantic rather than perceptual distinctiveness. Thus, when items are from different categories, the effect hardly appears even if they are perceptually similar (Schmidt et al., 2020). Therefore, in the present study, we tested how the passage of time influenced mnemonic discrimination as a function of the number of similar traces stored in memory. Accordingly, we varied the number of items encoded from each category (two and six items) and evaluated recognition at two different delays (20 min and 24 h). Additionally, we recorded brain activity using an EEG system with 128 electrodes while participants performed the recognition phase of the task.

We expect that the ability to discriminate between old and similar objects in both groups would decrease as the encoded number of items increases (Konkle et al., 2010; Poch et al., 2019). In view of previous results showing that details tend to fade over the passage of time, we also expect an increase in gist-based false recognition with longer delay (24-h delay). However, the ability to discriminate between old and novel objects will increase as the encoded number of items increases and would not be affected by the length of the delay. We also considered that these effects would be reflected in the modulation of event-related potentials (ERPs) associated with recognition processes.

Accumulating evidence has supported that recognition is the result of the contribution of two different processes: familiarity and recollection. The former is related to the idea that recognition can take place based on a general sense that an item has been previously encountered without retrieval of contextual details. Recollection implies that recognition of the probe is accompanied by the retrieval of specific details of the encoding episode (Yonelinas, 2002). Remarkably, these processes can be dissociated at their neural basis and, accordingly, have different ERP signatures (Yonelinas, 2002; Rugg and Curran, 2007; Skinner and Fernandes, 2007). In this sense, familiarity is associated with a negative going modulation recorded over mid anterior scalp locations between 300 and 500 ms, termed the FN400. Recollection is associated with a positive going modulation recorded over posterior parietal scalp locations between 500 and 800 ms, termed the parietal old/new effect (Curran et al., 2006; Rugg and Curran, 2007). When considering the ERP data, we expected that the behavioral results in the 20-min group would modulate the old/new effects in familiarity (FN400) and recollection (parietal old/new effect) time windows. As the higher encoded number of items increases the sense of familiarity, the FN400 amplitudes for old and similar items would be less different. In addition, the overlap between similar memories as the encoded number of items increases would place greater demand on pattern separation processes (Anderson et al., 2017). In this way, parietal old/new effect amplitudes for old items would be larger than those for similar items due to the fine discrimination between them through pattern separation (Anderson et al., 2017). Other research has previously shown that ERP amplitudes are attenuated as delay increases. Thus, we expected that ERP amplitudes will be attenuated in the 24 h group. Specifically, FN400 amplitudes for old and similar items would be less different, but also for old and new items. Additionally, parietal old/new effect amplitudes for old items would differ less from those for similar items.

## Methods

### Participants

Fifty-one students of the Universidad Autónoma de Madrid, with no medical history of neurological or psychiatric diseases, participated in the study for course credit. Participants were divided into two groups, one group completed the recognition phase 20 min after the study phase (Group 1), while the other group completed the recognition phase 24 h after the study phase (Group 2). The mean age of the participants in group 1 (16 females and 10 males) was 19.92 years (SD = 1.32). The mean age of the participants in group 2 (22 females and 3 males) was 19.43 (SD = 1.59). All participants signed an informed consent detailing the procedures of the study in accordance with the Declaration of Helsinki (1991). The study was approved by the Local Ethical Committee.

### Stimuli and Procedure

**Figure 1:**
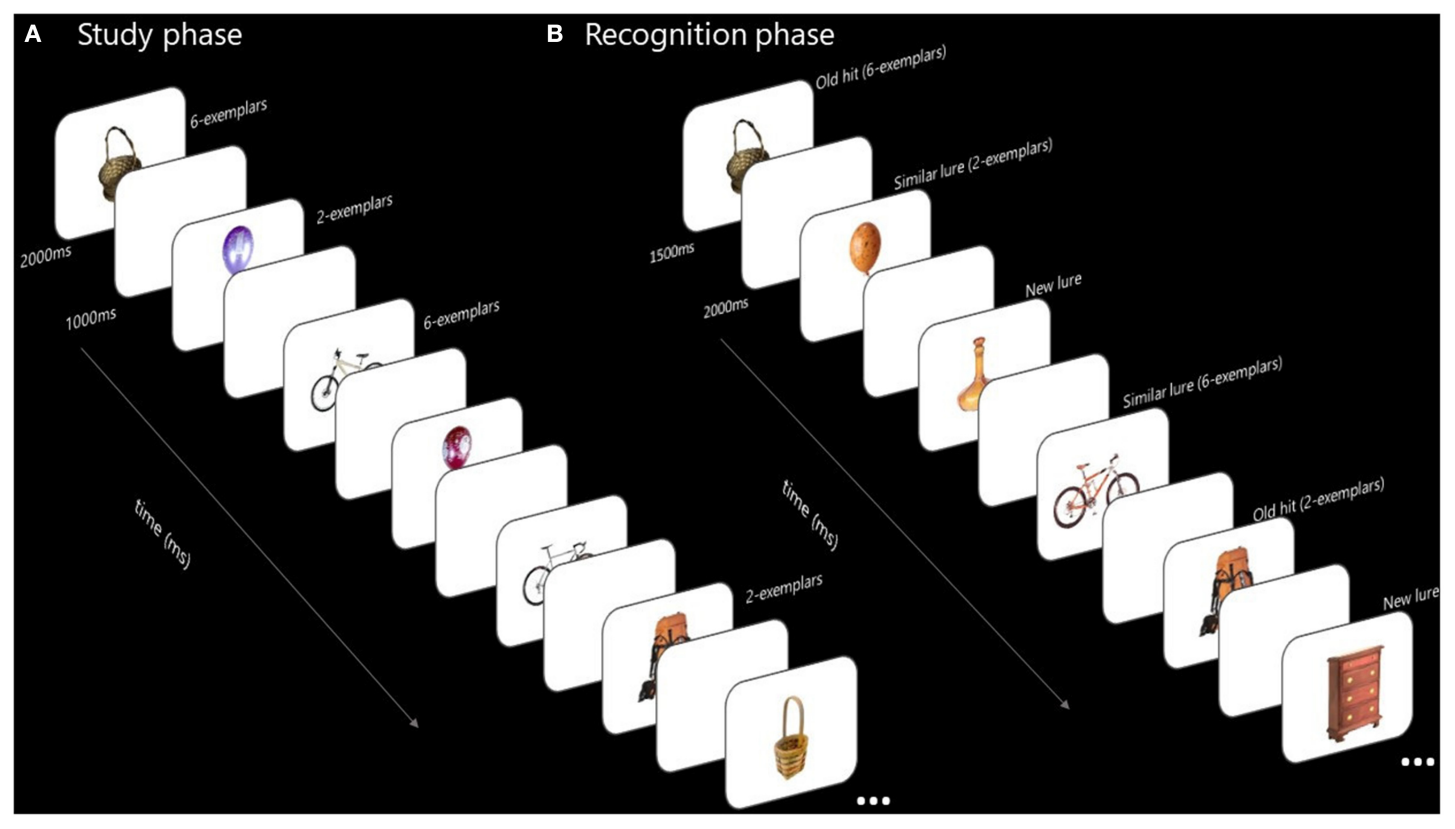
Test structure and examples of the items employed. The 1,440 images of objects from different categories were presented at the study phase. Each image was presented for 1,500 ms, followed by a 1,000 ms duration white screen. The number of images presented for each category was manipulated to 2 or 6 items. Twenty minutes (in group 1) or 24 h (in group 2) after the study phase, discrimination phase was tested. The 540 images of objects from different conditions (old, similar, or new) were presented for 1,500 ms, followed by a 2,000 ms duration white screen. Participants had to indicate if each of the stimulus presented: had been previously seen in the study phase (old), were a new image of an object not previously presented but from a category previously seen in the study phase (similar) or were an image of a new object from a new category (new). **(A)** Study phase. **(B)** Recognition phase.

A mnemonic discrimination task with study/test format was used (Stark et al., 2019). The stimuli and procedure (see Figure 1) were adapted from those reported by Poch et al. (2019). In the study phase we presented 1,440 images of objects of different categories (pictures did not show human body parts or animals), and varied the number of items from each category, specifically 2 or 6 items. Therefore, each participant saw 180 categories of two Item condition (360 pictures) and 180 categories of six Item condition (1,080 pictures). Each picture was presented for 1,500 ms, followed by a white screen (1,000 ms). There were 4 blocks (360 pictures for each block), and participants were allowed to take an untimed rest at the end of each one. Participants were instructed to remember all the images.

Participants in group 1 took a 20-min break after all the images had been presented. After the 20-min break they went through the discrimination phase, which consists in a random presentation of 540 pictures: 180 old items (pictures they had seen in study phase), 180 lures items (pictures from the same object category and similar to the old item, but not identical to those previously seen) and 180 new items (foils, pictures of non-presented categories) (Stark et al., 2019). Old and lure pictures were drawn equally from the 2- and 6-item conditions. Each picture was presented for 1,500 ms followed by a white screen (2,000 ms). Participants had to respond while the picture is on screen if it is an old item (pressing the key number 1), a similar item (pressing the key number 2) or a new item (pressing key number 3). There was one untimed break in the middle of the study phase. We tested the first object presented from each category to ensure the effects were due to stimulus interference (Konkle et al., 2010). Participants in group 2 went through the same discrimination test, but 24 h after the study phase.

### EEG Acquisition and Processing

Data were acquired during the discrimination phases of the task using a Biosemi Active Two system with 128 electrodes. Additional EOG-vertical and horizontal-electrodes and a tip-nose reference were also recorded. Online referencing was to sensors located in the posterior area of the cap (CMS and DRL). Moreover, these data were digitalized at a sampling rate of 2,048 Hz. Data were offline re-referenced to the nose tip and down-sampled to 256 Hz in Matlab using Fieldtrip software package (20201113 version; www.fieldtriptoolbox.org), a toolbox implemented in the Matlab environment (R2019b version; The MathWorks, Natick, MA). We included old hits (correct responses for old images), similar CR and new CR (correct rejections for similar and new images), and similar FA (similar false alarms, which are similar lures attracting false “old” responses), in the analysis following Morcom (2015) study. The continuous sets of raw data were segmented into −500 to 1,200 ms epoch around the presentation of each trial. After the segmentation, an infomax independent components analysis was performed to eliminate the horizontal eyes movements and blinks. Finally, epochs contaminated with other artifacts were rejected with a visual inspection criteria. The signal was down pass filtered with a low cut-off at 30 Hz and averaged separately for each condition and participant. ERPs were then baseline corrected (−200 ms). ERPs of all conditions and participants were then averaged.

All data were collected in accordance with the security measures for COVID-19, which include use of approved masks by both participants and researchers, cleaning hands with alcoholic gel before and after the experiment by both participants and researchers, sanitation of the data collection cabins, and material used and 10-min ventilation between participants. No case of contagion was reported by the participants.

Five participants were discarded due to technical problems during recording. There was a total of 21 participants in the 24-h group and 25 in the 20-min group. The mean numbers of trials (total and range in brackets) contributing to individual subjects' ERPs in the 24-h group in each condition were 27 (574, 13–56) to old hits from 2-item condition, 28 to similar CR from 2-item condition (595, 2–49), 41 to old hits from 6-item condition (877, 18–71), 31 to similar CR from 6-item condition (661, 10–63), 127 to new CR (2,675, 49–167), 11 to similar false alarms from 2-item condition (231, 2–29), and 35 to similar false alarms from 6-item condition (742, 12–77). The mean numbers of trials (total and range in brackets) contributing to individual subjects' ERPs in the 20-min group in each condition were 29 (732, 12–43) to old hits from 2-item condition, 35 to similar CR from 2-item condition (893, 13–60), 38 to old hits from 6-item condition (966, 20–57), 30 to similar CR from 6-item condition (772, 12–46), 124 to new CR (3,114, 81–168), 10 to similar false alarms from 2-item condition (272, 2–28), and 39 to similar false alarms from 6-item condition (994, 15–64).

### Statistical Analyses

#### Behavioral Data

Like previous studies, dependent variables of interest were correctly identified items and similar lure false alarms (Toner et al., 2009; Morcom, 2015). Specifically, dependent variables were correctly identified targets (Old hits), correctly rejected lures (Similar CR), correctly rejected foils (New CR), and incorrectly identified lures (Similar FA). We used a series of mixed-design analysis of variance models (ANOVAs) for Old hits, Similar FA and Similar CR, where Delay is a between-subject factor with two levels (20-min delay and 24-h delay) and Item is a within-subject factor with two levels (2 items and 6 items). For New Correct rejections we used an one-way ANOVA with Delay is a between-subject factor. After the ANOVA tests, we used the Bonferroni corrected pairwise comparisons to determine which means were significantly different. Analyses were performed using IBM SPSS Statistics 21.0 for Windows. We repeated these analyses for the time reaction.

Following Toner et al. (2009), we calculated a discrimination performance index which was calculated by subtracting the number of correct rejections to similar lures from the number of false alarms to similar lures. We used a repeated measures ANOVA with Group (20-min delay and 24-h delay) as the between-subject factor and Item (2 and 6) as the within-subject factor.

#### EEG Data

Statistical analyses of the ERPs were performed using a non-parametric cluster-based random permutation analysis approach (Maris and Oostenveld, 2007), in the two windows of interest (300–500 ms and 500–700 ms). This approach is an unbiased statistical that allows the identification of the spatial distribution of statistical effects, while effectively handles the multiple-comparisons problem. Specifically, permutation tests were used to compute the sampling distribution of a cluster-based statistic. Cluster-based statistics consist in grouping together spatial and temporal adjacent variables (*t*- or *F*-values for instance) into clusters. The cluster statistic can be defined by its maximal value, extension or a combination of both (Maris and Oostenveld, 2007). The analytic steps used here were as follows. First, a parametric statistical test was performed at each electrode in the averaged window of interest. *P*-values below 0.05 were used to form clusters of adjacent electrodes. A minimum of two channels were used to form a cluster. Cluster-level test statistic was calculated by taking the sum of all the individual t-statistics or *F*-values within that cluster. Then, a null distribution was created by computing 1,000 randomized cluster-level test statistics. Finally, the observed cluster-level test statistics was compared against the null distribution and only clusters falling above the 95th percentile were considered significant.

We used an independent samples test for the delay-group condition to determine whether there were statistically significant differences between the means of the two groups. We used a one-way repeated measures ANOVA for Category condition to determine whether there were any statistically significant differences between the means of the four levels (old hits, similar CR, similar FA, and new CR). We used a dependent-sample test for the Item condition to determine whether there were statistically significant differences between the means of the two levels (2 and 6). We used an independent-sample test for the interaction between factors.

We also performed an analysis separating the EEGs in terms of delay groups. This way, we used a one-way repeated measures ANOVA for Category condition to determine whether there were any statistically significant differences between the means of the four levels (old hits, similar CR, similar FA, new CR) in the 20-min group and we repeated the same analysis for the 24-h group. We also used a dependent-sample test for the Item condition to determine whether there were statistically significant differences between the means of the two levels (2 and 6) in both groups.

## Results

### Behavioral Results

#### Mnemonic Discrimination

For the Old hit responses, the ANOVA showed a significant main effect of Item [*F*_(1,49)_ = 124.65, *p* < 0.001, ${\text{η}}_{\text{p}}^{2}$ = 0.718]. This effect was explained by a higher proportion of Old hit responses in the 6-item condition (M = 51.59; SD = 14.81) than in the 2-item condition (M = 37.97; SD = 13.49). The ANOVA also revealed a significant interaction of Delay by Item [*F*_(1,49)_ = 5.04, *p* < 0.05, ${\text{η}}_{\text{p}}^{2}$ = 0.09]. Bonferroni pairwise comparisons indicated that Old hit responses were higher when 6 items had been stored [(M = 49.54; SD = 10.80), (M = 53.71; SD = 18.05), for 20 min and 24 h delay, respectively] compared to when 2 items had been stored [(M = 38.62; SD = 11.62), (M = 37.29; SD = 15.41), for 20 min and 24 h delay, respectively] in both delay groups (all *p*s < 0.001).

For Similar FA, the ANOVA yielded a significant main effect of Item [*F*_(1,49)_ = 299.24, *p* < 0.001, ${\text{η}}_{\text{p}}^{2}$ = 0.859]. This effect was explained by a higher proportion of Similar FA in the 6-item condition (M = 45.18; SD = 16.99) than in the 2-item condition (M = 13.18; SD = 8.79).

For Similar CR, the ANOVA revealed a significant Delay by Item interaction [*F*_(1,49)_ = 6.35, *p* < 0.05, ${\text{η}}_{\text{p}}^{2}$ = 0.115]. Bonferroni pairwise comparisons showed that the proportion of similar CR responses was higher in the 2-item condition (M = 44.274; SD = 15.104) than in the 6-item condition (M = 37.969; SD = 10.401) in the 20-min group.

For New CR, the ANOVA did not show any significant result.

Interestingly, none of the ANOVAs revealed a significant main effect of Delay (all *p*s >0.20).

**Figure 2:**
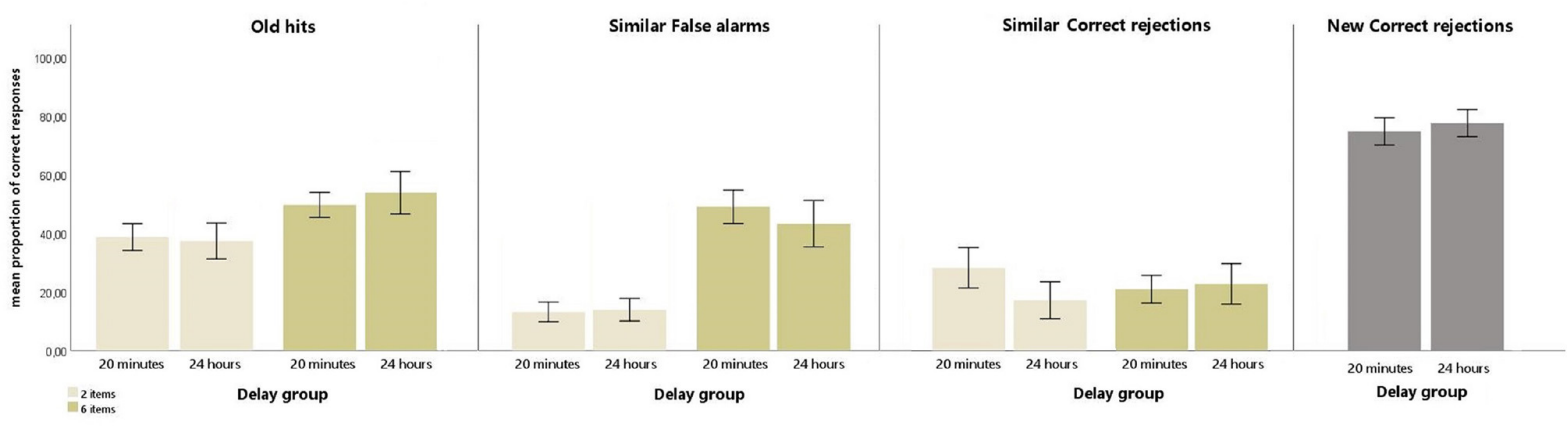
The mean proportion (and standard errors) of responses of Old hits, Similar correct rejections, and Similar false alarms of 2 and 6 items with a 20-min delay and a 24-h delay, and the mean proportion (and standard errors) of responses of New correct rejections with a 20-min delay and a 24-h delay.

Figure 2 depicts the mean proportion of responses for each type of response.

**Figure 3:**
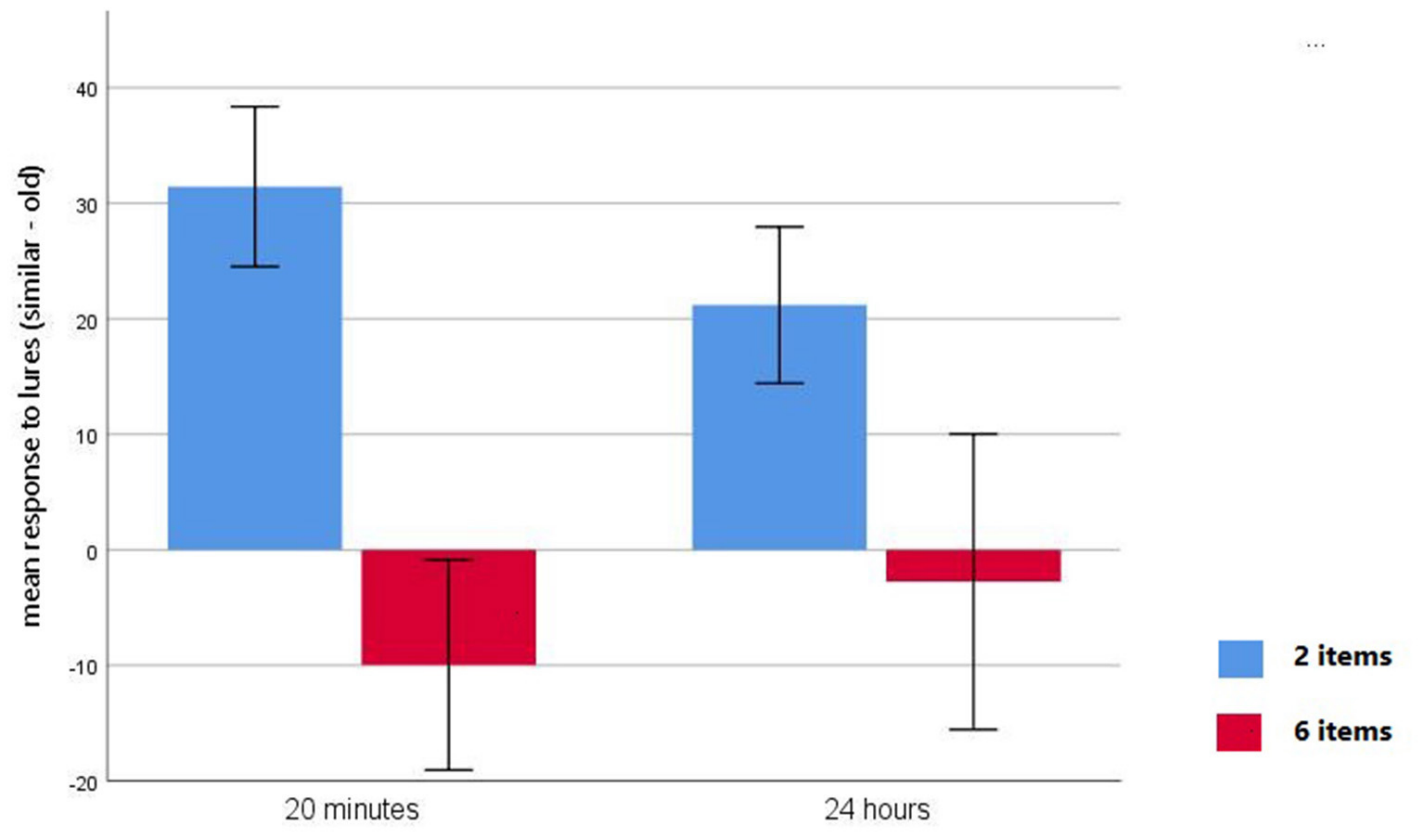
Mean difference scores (number of similar correct rejections minus number of similar false alarms) for 2-item condition (blue bars) and 6-item condition (red bars) with a 20-min delay and a 24-h delay.

Analysis of the discrimination performance index yielded a main effect of Item [*F*_(1,49)_ = 75.05, *p* < 0.0001]. Bonferroni pairwise comparisons indicated that participants exhibited a higher discrimination capacity in the 2-item condition. Analysis also showed a Group by Item interaction [*F*_(1,49)_ = 5.39, *p* < 0.05]. Bonferroni pairwise comparisons revealed that there were significant differences in the discrimination index between delay groups in the 2-item condition, with participants in the short-delay condition showing higher discrimination capacity than those in the long-delay condition (M = 31.44, SD = 17.14 for 20-min, M = 21.19, SD = 16.40 for 24 h; *p* < 0.05). No significant differences were observed between delay groups in the 6-item condition (M = −9.97, SD = 22.51 for 20-min, M = −2.76, SD = 31.01 for 24 h; *p* >0.30; Figure 3). Pairwise comparisons also indicated that in both delay groups the discrimination index was higher in the 2-item condition than in the 6-item condition (all *p*s < 0.001; Figure 3).

#### Reaction Time

The mixed ANOVA showed there was a significant Category main effect [*F*_(2.160, 105.862)_ = 53.336, *p* < 0.01, ${\text{η}}_{\text{p}}^{2}$ = 0.521]. Bonferroni pairwise comparisons showed a significant difference between similar CR and all other conditions: similar CR vs. old hits (*p* < 0.01), similar CR vs. similar FA (*p* < 0.01) and similar CR vs. new CR (*p* < 0.01). The reaction time was always slower for similar CR (M = 1.096; SD = 0.096) than for old hits (M = 0.991; SD = 0.085), similar FA (M = 0.993; SD = 0.93) or new CR (M = 0.955; SD = 0.104). This pattern of behavior was replicated in both groups, so we found there was not a significant interaction between Category by Delay.

The second mixed ANOVA showed a significant Category main effect [*F*_(1.577, 77.262)_ = 34.638, *p* < 0.01, ${\text{η}}_{\text{p}}^{2}$ = 0.414]. Bonferroni pairwise comparisons showed a significant difference between similar CR and old hits (*p* < 0.01), and between similar CR and similar FA (*p* < 0.01). The similar CR condition was significantly different from the other two conditions in both groups, and also in both Item conditions, so there was not a significant interaction between Item by Category by Delay.

### ERPs Results

Non-parametrical statistics revealed that event-related potentials amplitudes were significantly higher in the 20-min group than in the 24-h group in the two time-windows of interest. In the first window (300–500 ms), there was a more positive activity in a central-anterior cluster of electrodes for the 20-min group compared to the 24-h group, especially on the right side (*p* < 0.05). In the second temporal window (500–700 ms), the more positive activity in the 20-min group was widely distributed across the scalp (*p* < 0.01). Statistical analysis of the Item main effect revealed greater ERP amplitude in the 6-item condition compared to the 2-item condition in the late temporal window (500–700 ms) in a cluster of centroposterior electrodes (*p* < 0.05).

Differences between Category conditions were significant in the 500–700 ms time window in centroparietal electrodes, especially on the left-side (*p* < 0.05). *Post-hoc* analysis revealed larger ERP amplitudes for old hits compared to similar CR and new CR (*p* < 0.05 Bonferroni corrected) and for similar FA compared to new CR (*p* < 0.05 Bonferroni corrected).

The cluster non-parametrical approach allows us to test the statistical significance of the interaction between two factors. Consequently, the interactions of Group and Item, Group and Category, and between Item and Category, were tested without obtaining any significant effect (*p* > 0.05). This statistical approach overcomes the problem of multiple testing, but can be conservative in many cases. In this experiment, although Group interactions were not significant, visual inspection of the data revealed a different ERP modulation as a function of Group. For this reason, we performed independent statistical analysis of the main factors Category and Item for each of the Groups.

**Figure 4:**
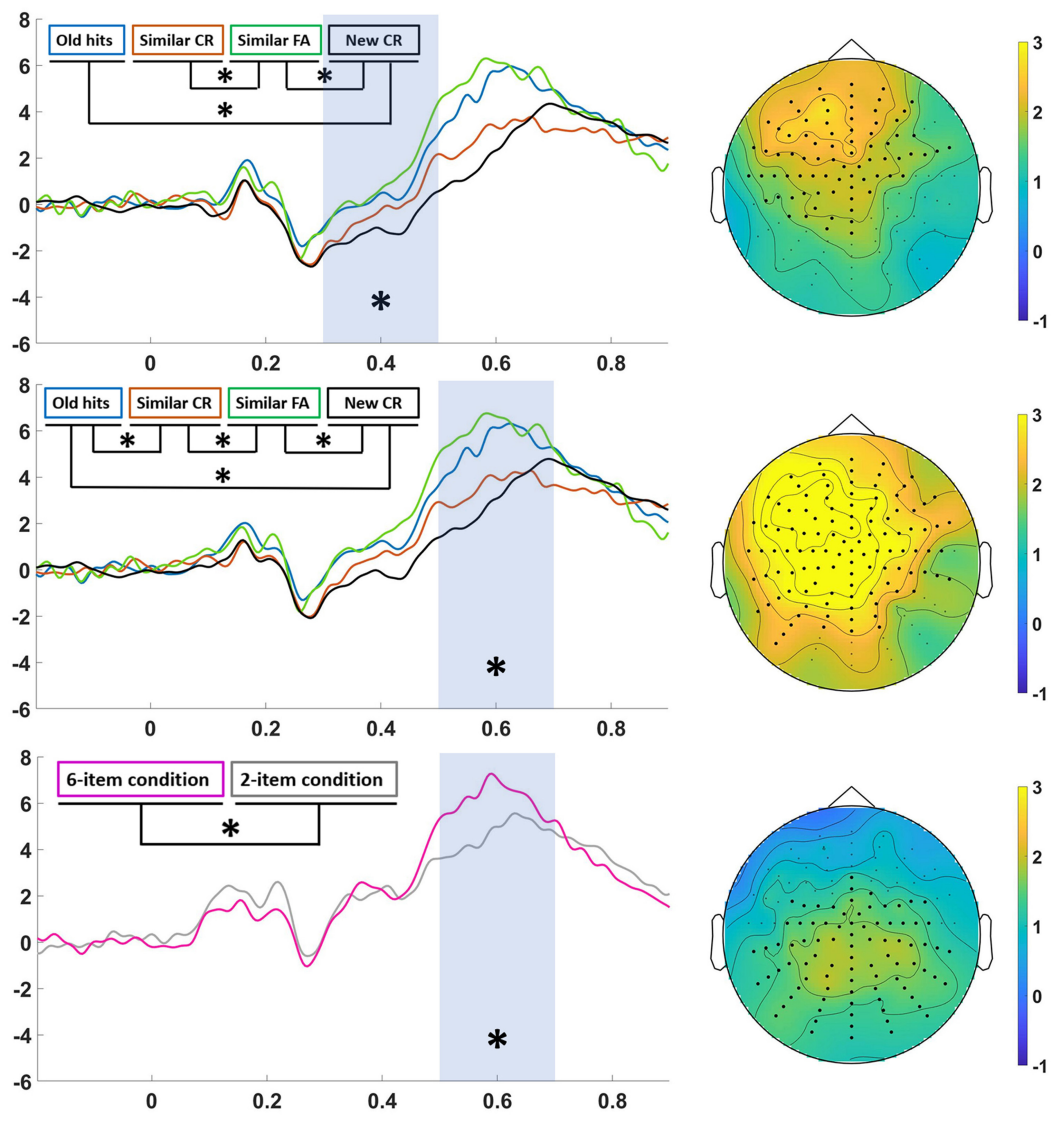
Analysis in the 20-min group. Significant differences are marked with an asterisk in the ERPs and with black circles in the topography. Left: ERPs from “old hits” (blue line), “similar CR” (red line), “similar FA” (green line) and “new CR” (black line) in the 300–500 ms (top) and 500–700 ms (middle) time window, and ERPs from “6-item condition” (pink line) and “2-item condition” (gray line) in the 500–700 ms time window (down). Right: topographic map of the grand-average of “similar FA-new CR” in the 300–500 ms time window (top), “similar FA-new CR” (middle) in the 500–700 ms time window (middle), and “6-item condition-2-item condition” in the 500–700 ms time window (down).

Statistical analysis in the 20-min group revealed a significant Category main effect in the earlier window (300–500 ms) in an anterior-central area (*p* < 0.01) and in the later window (500–700 ms) in a cluster of central electrodes (*p* < 0.01). In both windows, ERPs corresponding to old hits and similar FA were significantly greater than new CR (*p* < 0.05 Bonferroni corrected). In the early window similar FA were also greater than similar CR (*p* < 0.05 Bonferroni corrected), and in the later window both old hits and similar FA were also significantly greater than similar CR (*p* < 0.01 Bonferroni corrected). We also found a significant Item main effect in the 500–700 ms time window located in a posterior area (*p* < 0.05) (especially on the left side), in which ERP amplitudes were larger for Item 6 than for Item 2 (Figure 4, down). We found a non-significant interaction between Category and Item factors.

The same statistical analysis was conducted for the 24-h Group. Critically, we found no cluster of ERP differences for the main effect of Category or Item.

## Discussion

Theories proposing that the forgetting of episodic events can be explained by interference processes have gained further support in the last years (Hardt et al., 2013). Accumulated evidence typically revealed greater mnemonic discrimination failures as the similarity between memory events increases (Stark et al., 2019), and as the number of similar traces increase (Roediger and Agarwal, 2010; Anderson, 2015; Poch et al., 2019). In the present study, we investigated memory discrimination between similar events as a function of passage of time, and, for the first time, as a function of the number of similar traces stored in memory increases. To test these hypotheses, we administered a visual mnemonic discrimination task to two groups of young healthy participants in which we parametrically manipulated the number of items studied from each category, using two different recognition delays. This allowed us to dissociate the effects of number of similar traces and the passage of time over mnemonic discrimination. We also explored ERP amplitudes while participants performed the visual mnemonic discrimination task. To our knowledge this is the first time that similarity, number of stored items, and passage of time have been considered together in the context of a mnemonic discrimination task.

### Behavioral Findings

We found that the number of events stored in memory modulated the ability of the participants to correctly identify a previously studied item (“old hit”). This is, participants were more accurate when the number of studied objects from the same category increased. These findings suggest that the more semantically related information we study, the more we increase our recall of that category. In contrast, we observed that participants committed more similar false alarms as the number of items increased (i.e., four times more for the 6-item condition than for the 2-item condition), which could be considered as a gist-based false recognition (Guerin et al., 2012a,b). These findings suggest that the accumulation of similar memories strengthened the familiarity of the object category (Konkle et al., 2010; Poch et al., 2019; Stark et al., 2019; Zotow et al., 2020), favoring the identification of previously encountered items, but decreased the ability to discriminate between similar and old items (Guerin et al., 2012a). In other words, there is a shift toward generalization (i.e., pattern completion), that occurs at the expense of discrimination (i.e., pattern separation; Yassa et al., 2011). This may indicate that, at retrieval, participants relied on a more abstract representation or gist-based processing when more items had been stored in memory, while relied on a more detailed memory representation when fewer items had been stored in memory (Schacter et al., 2011). As we found no significant differences between the delay-groups, it seems that mnemonic discrimination was not modulated by the passage of time, but by the number of stored events (Schacter et al., 2011). The results of the discrimination index indicated that this was the case, since participants exhibited higher lure discrimination ability when fewer items had been stored in both delay groups (Figure 3). Some previous studies have suggested that the fade of detailed memories over time might had been overestimated (Guerin et al., 2012b; Andermane and Bowers, 2015). It is also possible that longer delays are needed to observe a decline in memory performance (Roberts et al., 2013; Wang, 2014; Andermane and Bowers, 2015; Tsivilis et al., 2015; Mercer and Jones, 2019). Nonetheless, we found that similar correct rejections were higher in the 20-min delay group than in the 24-h delay group when two items were stored in memory. It could be thought that, when fewer items are stored in memory, and there is less interference, passage of time most likely induces discrimination failures (Leal et al., 2019). This conclusion is supported by the observation that the discrimination index was higher in the 20-min delay group than in the 24-h delay group when two items were stored in memory.

In addition, mean response times of participants were slower when they correctly classified a similar object than an old one. The mean response was also slower for the similar CR than the similar FA. These findings partially agree with Morcom (2015), who also found that false alarms to similar items were faster than similar correct rejections. On the contrary, we found no significant differences between similar FA and old hits, while Morcom found that hit responses were the fastest.

### ERP Findings

Analyses of brain activity revealed several findings. First, we found a Group main effect in both the 300–500 and the 500–700 ms time windows, where there was a greater positive activity for the group that performed the recognition phase 20 min after encoding than the group that performed the recognition phase 24 h. Previous studies have shown a reduced amplitude in posterior regions between 500 and 700 ms when comparing short and long delays (Roberts et al., 2013; Tsivilis et al., 2015), but not in the 300–500 ms time window. Based on a behavioral experiment, these results were interpreted as indicating that recognition after longer delays was based on familiarity. Similar to these studies, Curran and Friedman (Curran and Friedman, 2004) did not found differences between short and long delays between 300 and 500 ms. As we did not find an impact of time delay in discrimination (Curran and Friedman, 2004), it is difficult to interpret ERPs differences. It might be possible that passage of time reduces the amplitude of ERPs, but this attenuation only modulates discrimination accuracy after a certain period of time (Roberts et al., 2013).

We also found that increasing the number of exemplars presented from a category modulated ERP amplitudes, so that participants showed a more positive activity in a centro-posterior region in the 500–700 ms window at retrieval when more items had been stored in memory. Considering that as items in memory increases interference is higher and performance is worse, this late posterior effect might be related with more resources recruited when it was harder to perform discrimination decisions (Poch et al., 2019).

Finally, analyses also yielded a main Category effect in the 500–700 ms window. Waveforms related to correctly recognized studied items (i.e., “old hits”) and falsely recognized lures (i.e., “similar FA”) did not differ between them. Interestingly, ERP amplitudes for old hits and similar FA were greater than ERP amplitudes for correctly rejected new items (Roberts et al., 2013; Morcom, 2015; Tsivilis et al., 2015; Cadavid and Beato, 2016). These differences were found over a centro-posterior region, the so-called parietal old/new effect (Curran and Cleary, 2003; Curran et al., 2006; Rugg and Curran, 2007; Morcom, 2015). This parietal old/new effect indicates that a recollection-based process was observed not only in correct recognition, but also in false recognition (Goldmann et al., 2003). As previously stated (Morcom, 2015; Cadavid and Beato, 2016), these findings support the view that false recollections are at the base of false memories. This effect has been previously linked to pattern completion (Anderson et al., 2017). Previous studies have shown that a later frontal component differed between true and false recognition and has been interpreted as reflecting effortful post-retrieval processes (Goldmann et al., 2003; Wiese and Daum, 2006), although others have not found this difference (Cadavid and Beato, 2016). Crucially, amplitudes for Old hits were greater than amplitudes for similar CR (Morcom, 2015; Cadavid and Beato, 2016). This finding indicates that the processes associated with correctly recognizing a studied item and with avoiding a false recognition of related lures rely on different neural mechanisms (Cadavid and Beato, 2016). Interestingly, Johnson and colleagues found that waveforms for old and lure items could be modulated by test format, such that waveforms for true and false memories are more similar when items are randomly intermixed as compared to when they are presented using a blocked procedure (Johnson et al., 1997). Finally, we found that the waveforms for correct rejections of similar lures and the waveforms for correct rejections of new items were comparable (Anderson, 2016). The absent of differences between these two signals indicates that correctly rejected related lures lacked recollection (Cadavid and Beato, 2016). Interestingly, current results match the hypotheses in Anderson's studies (Anderson, 2016; Anderson et al., 2017), where they proposed that Similar CR reflects pattern separation processing, and then the associated ERP waveforms should resemble those of the New CR.

**Figure 5:**
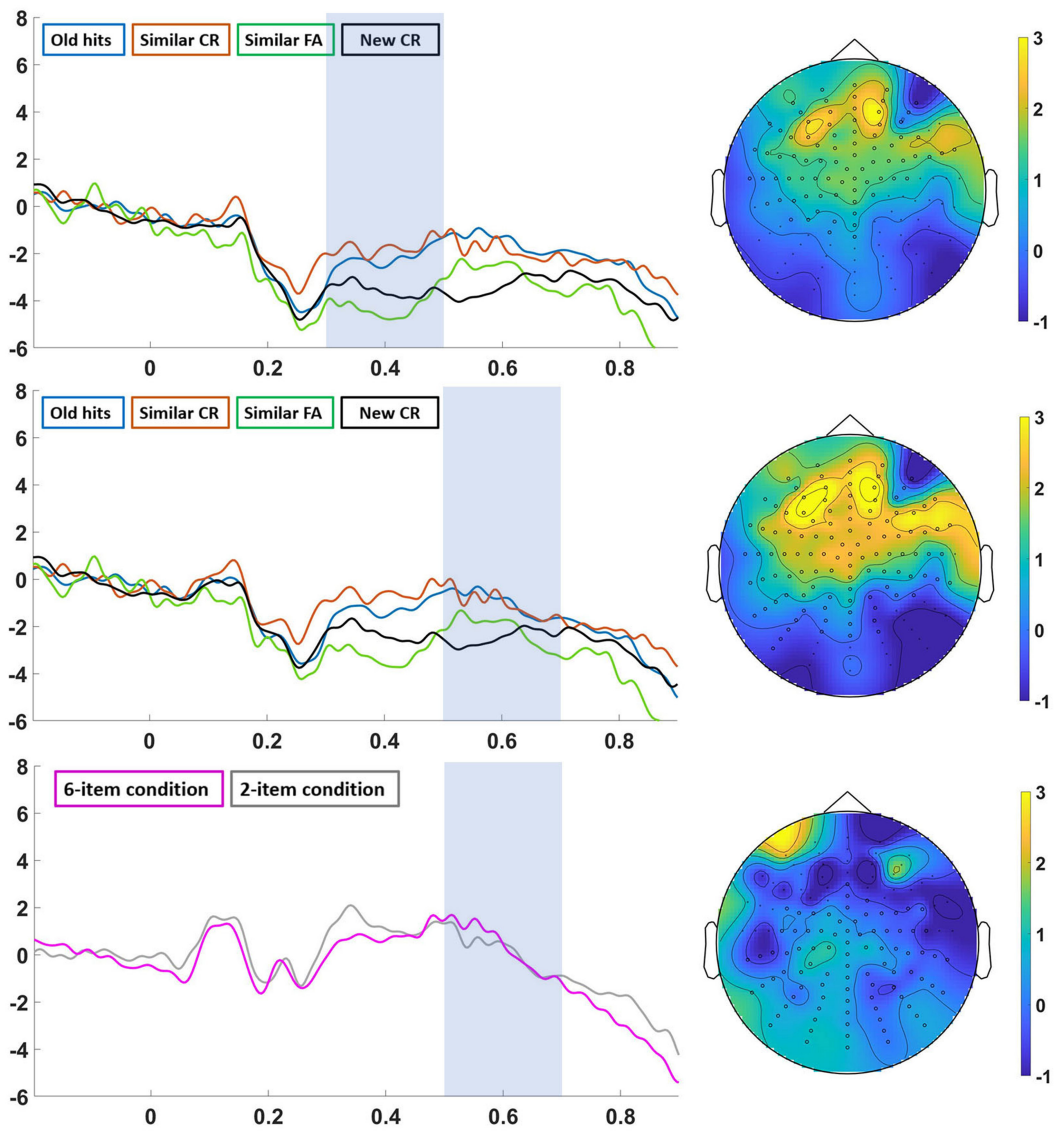
“Analysis in the 24-h group”. Left: ERPs from “old hits” (blue line), “similar CR” (red line), “similar FA” (green line) and “new CR” (black line) in the 300–500 ms (top) and 500–700 ms (middle) time window, and ERPs from “6-item condition” (pink line) and “2-item condition” (gray line) in the 500–700 ms time window (down). Right: topographic map of the grand-average of “old hits-new CR” in the 300–500 ms time window (top), “old hits-new CR” (middle) in the 500–700 ms time window (middle), and “6-item condition-2-item condition” in the 500–700 ms time window (down).

When we analyzed both delay groups separately, a different picture emerged (see Figures 4, 5). First, we found that the waveforms for all the categories of response were similar in both groups, although we only found significant differences in amplitude among them in the short-delay group. These results differed from previous studies reporting old/new effects after long delays (Curran and Friedman, 2004; Roberts et al., 2013; Cadavid and Beato, 2016). Similarly to the main analysis, we observed that true and false recognition showed an old/new effect in the 500–700 ms time window. In this case, ERP amplitudes for both type of responses were more positive than correct rejection of new items and correct rejection of similar lures (i.e., Similar CR; Wiese and Daum, 2006). Differently from the main analysis, we also found an effect in the 300–500 ms over an anterior area, the so-called familiarity-related component FN-400. Interestingly, true, and false recognition were more positive than correct rejection of new items. This effect points to a familiarity-based process on true and false recognition (Cadavid and Beato, 2016). Crucially, we also found that true and false recognition signals were more positive than correct rejected similar lures. This finding indicates that similar lures are associated with different familiarity processes depending on if they are falsely recognized or not (Brainerd and Reyna, 2002; Cadavid and Beato, 2016).

It is important to highlight that the limited number of trials in some of the conditions could have impacted the obtained results. Specifically, in the 2 items condition, False Alarms in 21 out of 45 subjects had less than 10 trials. Statistical differences of the main effects were calculated based on the average of all the trials of each Category or each Item, so the negative impact of fewer trials in only one condition is attenuated. However, ERPs calculated based in less than 10 trials is a limitation in the assessment of the interactions, in which the statistical significance is calculated based on each ERP condition.

## Conclusions

In summary, current results suggest that mnemonic discrimination is modulated by the interference associated with increased number of similar events stored in memory, rather than with the passage of time. Nonetheless, previous studies have reported a declining in mnemonic discrimination as a function of time. This discrepancy could be partially explained by the difference in delay rates used, which were much longer in the other experiments (Brady et al., 2013; Andermane and Bowers, 2015; Sekeres et al., 2016; Mercer and Jones, 2019). In view of the ERP results, current findings support the idea that amplitudes for recent and distant events are different, with an attenuation of amplitudes in longer delay (Roberts et al., 2013; Tsivilis et al., 2015). Additionally, we observed that both true and false recognition are based on familiarity- and recollection-based memory mechanisms (Brainerd and Reyna, 2002). Finally, our results also indicate that similar lures are associated with different familiarity processes depending on if they are falsely recognized or not (Brainerd and Reyna, 2002; Cadavid and Beato, 2016).

## Data Availability Statement

The raw data supporting the conclusions of this article will be made available by the authors, without undue reservation.

## Ethics Statement

The studies involving human participants were reviewed and approved by Ethical Committee of Autonomous University of Madrid. The participants provided their written informed consent to participate in this study.

## Author Contributions

PC designed the study, analyzed the data, and wrote the manuscript. CP analyzed the data and wrote the manuscript. LG-R evaluated the participants, analyzed the data, and wrote the manuscript. All authors contributed to the article and approved the submitted version.

## Funding

This work was supported by a grant from Fundación Tatiana Pérez de Guzman el Bueno to PC. LG-R was supported by a grant from the Regional ministry of research and education of the Community of Madrid and European Regional Development Fund to PC [H2019/HUM-5705]. This work was partially funded by the Ministerio de Ciencia, Innovación y Universidades under grant PID2019-111335GA-I00 to CP.

## Conflict of Interest

The authors declare that the research was conducted in the absence of any commercial or financial relationships that could be construed as a potential conflict of interest.

## Publisher's Note

All claims expressed in this article are solely those of the authors and do not necessarily represent those of their affiliated organizations, or those of the publisher, the editors and the reviewers. Any product that may be evaluated in this article, or claim that may be made by its manufacturer, is not guaranteed or endorsed by the publisher.
